# Correlations between Colonization of Onion Thrips and Leaf Reflectance Measures across Six Cabbage Varieties

**DOI:** 10.1371/journal.pone.0073848

**Published:** 2013-09-05

**Authors:** János Bálint, Balázs Vince Nagy, József Fail

**Affiliations:** 1 Department of Entomology, Faculty of Horticultural Science, Corvinus University of Budapest, Budapest, Hungary; 2 Department of Horticulture, Faculty of Technical and Human Sciences, Sapientia University, Tîrgu-Mureş, Romania; 3 Department of Mechatronics, Optics and Engineering Informatics, Budapest University of Technology and Economics, Budapest, Hungary; 4 Institute of Psychology, University of Sao Paulo, Sao Paulo, Brazil; Volcani Center, Israel

## Abstract

The main purpose of this study was to reveal if the UV-A, and visible light reflection of leaves of white cabbage varieties is correlated to resistance against onion thrips. The antixenotic resistance (AR) against onion thrips and thrips damage differed between varieties Balashi, Bloktor, Riana – considered resistant – and Green Gem, Hurricane, Quisor – considered susceptible. The solar UV-A (340–400 nm) and visible (401–650 nm) light reflection of white cabbage leaves were recorded. Correlation between AR against onion thrips and reflection of leaves in UV-A and visible range of the studied white cabbage varieties were computed. According to the AR evaluation onion thrips density was always higher on susceptible than on resistant varieties. The UV-A light reflection of head forming leaves and the contrast between head and exterior leaves (H/E) was negatively correlated with onion thrips host preference at an early stage of cabbage head formation. The visible light reflection of both head forming and exterior leaves was also negatively correlated with onion thrips host preference. Susceptible varieties had greater damage ratings at harvest than resistant ones and positive correlations were observed between AR and damage. AR against onion thrips may be affected by differences in reflection of cabbage leaves at an early growth stage. It is suggested that more intensive reflection of leaves and/or higher contrast values between the reflectance intensity of head versus outer leaves made the resistant varieties less attractive to onion thrips. Our results reported here provide the first evidence of negative correlation between UV-A and visible reflection of leaves and AR of white cabbage against a dangerous insect pest, opening new perspectives for understanding the role of reflection by plant leaves in pest management.

## Introduction

One of the most important pests of cabbage is the onion thrips (*Thrips tabaci* Lindeman, 1889) [1], [2]. In the last two decades this pest has become a major problem in the summer production period [3]. Damage is produced by individuals from head forming leaves, here for susceptible varieties thrips number was twice high as on exterior leaves [4]. The most common method to control onion thrips populations is the use of foliar insecticides, but *T. tabaci* is difficult to control because insects are found mainly in the narrow spaces between the inner leaves [5]–[7]. The use of natural enemies (predatory mites) does not give adequate control either [8]. Other authors have stated that the primary control of onion thrips damaging cabbage may be the selection of tolerant varieties [9], [10]. Only a few experiments have been carried out in order to assess the mechanism that determines resistance of white cabbage against onion thrips. The term antixenosis was proposed by Kogan and Ortman [11] and defines host plant features (for instance leaf structures, light reflectance, chemical volatiles) that can positively or negatively influence the colonization of phytophagous insects. In later studies Fail et al. [12] confirmed that antixenosis plays a role in the resistance of white cabbage against onion thrips and also proposed that the leaf reflectance might have a significant role in host acceptance of onion thrips. Thus, thrips might use visual cues in deciding whether to colonize a cabbage-plant.

Insects select their host plant by visual cues between a plant and its environment [13]. For host plant recognition the most important factors are the hue (dominant wavelength reflection) the saturation (hue clearness) and the brightness [13]. Insects possess photoreceptors in their eyes that are sensitive to limited intercepts (colors) of the wavelength spectrum of solar radiation [14]. UV perception in the range of 350–390 nm is important for host plant detection by most insects [15], [16]. Experiments revealed that for many thrips species the UV reflection of surfaces between 350–370 nm were repellent [13]. UV sensitivity was assessed with electro-physiological methods on *Frankliniella occidentalis* Pergande, 1895 and with behavioral methods on *Caliothrips phaseoli* Hood, 1912 [17], [18]. Perception of UV was demonstrated for both species, UV-A sensibility of *F. occidentalis* and both UV-A and UV-B sensitivity of *C. phaseoli* were observed [17], [18]. A well-documented phenomenon is that the intensity of host plant consumption by insects frequently increases when the UV-B solar radiation is experimentally attenuated using filters [18]–[21] but the relationship between UV-A reflection of leaves and antixenotic resistance (AR) of cultivated plants against insects has not been previously addressed. Thus, thrips seem to respond to light reflectance properties, but the relationship between UV-A reflection of leaves and antixenotic resistance (AR) of cultivated plants against insects has not been previously investigated.

In this study the hypothesis that UV-A reflection may differ between white cabbage varieties was tested. More specifically the relationship between white cabbage leaves’ UV-A and visible reflectance,and AR against onion thrips was experimentally assessed.

## Materials and Methods

### Ethics Statement

All animal work was conducted according to relevant national and international guidelines. For insects collection no permits were required since the area where onion thrips were collected did not contain any strict protected areas, and onion thrips is not under protection in Europe. Also no permits were required to use insects for experiment due to the observational nature of the data collection. Formal agreements for experiment were obtained from Corvinus University of Budapest; the study was also agreed and supported by Institute for Ethnic and National Minority Studies of the Hungarian Academy of Sciences.

### Field Location and Experiment Design

Six white cabbage varieties were used in the experiment. Three of these varieties (Green Gem, Hurricane and Quisor) were considered susceptible, while the other three (Balashi, Bloktor and Riana) were considered resistant to onion thrips damage [3], [12]. Plants were raised at the Tordas Station of the Central Agricultural Office, Tordas, Hungary. Seedlings of 60×60 cm distance were transplanted in May, and two blocks for each variety were established in a randomized block design each with seven rows and 13 plants in each row (2×91 plants for each variety). Head formation started in June (Table 1).

**Table 1:** Start of head formation and timetable of evaluations.

Variety	Cupping starts		Antixenotic evaluation		Damage assessment	
	date	d.a.t.*	date	d.a.t.*	date	d.a.t.*
Balashi	10^th^ of June	26	25^th^ of June	41	11^th^ of August	88
Bloktor	18^th^ of June	34	25^th^ of June	41	10^th^ of October	148
Green Gem	10^th^ of June	26	19^th^ of June	35	12^th^ of August	89
Hurricane	13^th^ of June	29	24^th^ of June	40	17^th^ of September	125
Quisor	10^th^ of June	26	19^th^ of June	35	19^th^ of August	96
Riana	10^th^ of June	26	24^th^ of June	40	21^th^ of August	98

* d.a.t. = days after transplantation.

### Antixenotic Evaluation

We considered AR as the number of thrips colonizing white cabbage varieties at the beginning of the vegetation period during the cabbage head formation (cabbage head weight 34–114 g). Individuals already reproducing on cabbage head leaves must face biological properties too – antibiosis –, so the term antixenosis cannot be used at a later time, when newly emerged individuals on cabbage appeared. Samples consisted of 24 randomly selected cabbage heads from each block (a total of 48 sample/variety). Cabbage heads were kept in plastic bags at room temperature for a few hours in the laboratory until the antixenotic evaluation was completed. During the evaluation the first ten outer head leaves from every cabbage head were removed one by one and the number of adult thrips was counted on both upper and lower sides under a stereomicroscope. Resistance cannot be defined as an absolute value, (generally it is a relative feature) so rather than use the absolute number of colonizing thrips in the analyses, the proportion of total abundance (PTA) was considered. The total number of individuals from the first sample was separately summed from each variety and the proportion of total abundance (PTA) was computed. The same calculation was done for the next samples and the values gained were used as variables in statistical analyses.

### Leaves Reflection Measurements

Sample collections were made consistently for all varieties before sampling for the antixenotic evaluation. The number of replicates in each sampling event was 48 from each variety. Leaf sections of 7×7 cm were prepared from the exterior and first head-forming leaves of 12 plants of each variety. Leaf reflection was measured in the UV-A range from 340 to 400 nm with 1 nm spectral resolution. Additionally, the visible spectral reflections from 401 to 650 nm with 10 nm spectral resolution were recorded. Because the surfaces of the leaves were not homogenous (veins, damages, epicuticular wax, etc.) thereby causing differences in the spectral parameters, each leaf section was measured in four different locations. The measurements were carried out with two different instruments although with the same method. In both cases a standard white surface with known spectral reflection was measured prior to the leaf spectral reflections. This way the results were acquired in relative units. In the UV range we used a UV fluorescent lamp to irradiate the samples exchanging the standard white surface with the test samples. In the visible range this white calibration was included in the spectrophotometer, thus the comparison was done automatically. The measurement distances were chosen in a way so that the surfaces of the measured samples were significantly larger than the areas captured within the measurement angle of the instruments. With this adjustment also the background of the samples was neglected. Mean values of these four measurements were generated from all data points and used for analyses. Calibrated spectrophotometer (Konica Minolta CM-2600d) and spectroradiometer (AvaSpec-2048) were used to set up and accomplish the laboratory procedure for measuring leaf reflection in the UV-A and visible range. In case of spectroradiometric measurement the illumination of the leaves was provided by a specific fluorescent lamp, with significant UV-A emission between 340 and 400 nm.

### Onion Thrips Damage Assessment at Harvest

Assessment was carried out at harvest maturity for all varieties. Similarly to the antixenotic evaluation, 24 randomly selected cabbage heads were collected from each block. To estimate thrips damage an evaluation method was adapted using the methodology developed by Fail [3]. Cabbage head leaves were evaluated and peeled off one by one until no damage was found on four consecutive leaves. During this procedure the extent of damage was assessed for every examined head-forming leaf, expressing the proportion of damaged surface to the entire surface of the leaf. A scale from 0 to 1 with an accuracy of 0.1 was used where 0 means no damage and 1 means 100% damage. The cumulated value of damage ratios noted for every examined leaf in one cabbage head and the total number of damaged leaves in a given cabbage head was considered for data analysis.

### Data Analysis

A general linear model was used to test the block effects of PTA, total damaged leaf surface and number of damaged leaves using R language and environment. Residuals of the models were tested for normality and homogeneity of variance using package “geepack” geeglm function (Fit Generalized Estimating Equations - GEE) [22]–[24]. A similar general linear model was used to test the block effects of reflections using glm function [22]. PASW was used to test the proportion of total abundance and damage assessments. These data did not meet the assumption of normality, therefore the nonparametric Kruskal-Wallis test was used, followed by Mann-Whitney U tests to compare the varieties. For each variety in 12 external leaves the UV-A reflection between 340–400 nm was measured four times (n = 12 × average of 4 measurements/each variety). Then for each leaf a spectral average was computed from the reflection values of each nanometer between 340–400. In this way a sum of 12 average UV reflection values were obtained for each variety. These served as quantitative variable for statistical analyses. The same approach was followed to get the quantitative variable between 400–650 nm. The whole protocol was replicated for head forming leaves and the same variables in UV and visible range were obtained. To compare reflections the homogeneity of variances were tested with Levene test. UV-A data were not homogenous therefore the Games-Howell test was used while in visible range of light due to data homogeneity the Tukey HSD test was computed (PASW Statistics 18). Spearman’s rank correlation coefficient was calculated between mean values of reflection and PTA as well as between the contrast in reflectance of head forming and exterior cabbage leaves and PTA. Correlation between the number of adult thrips and damage assessed at harvest were similarly computed using PASW Statistics 18.

## Results

### Antixenotic Evaluation

**Figure 1:**
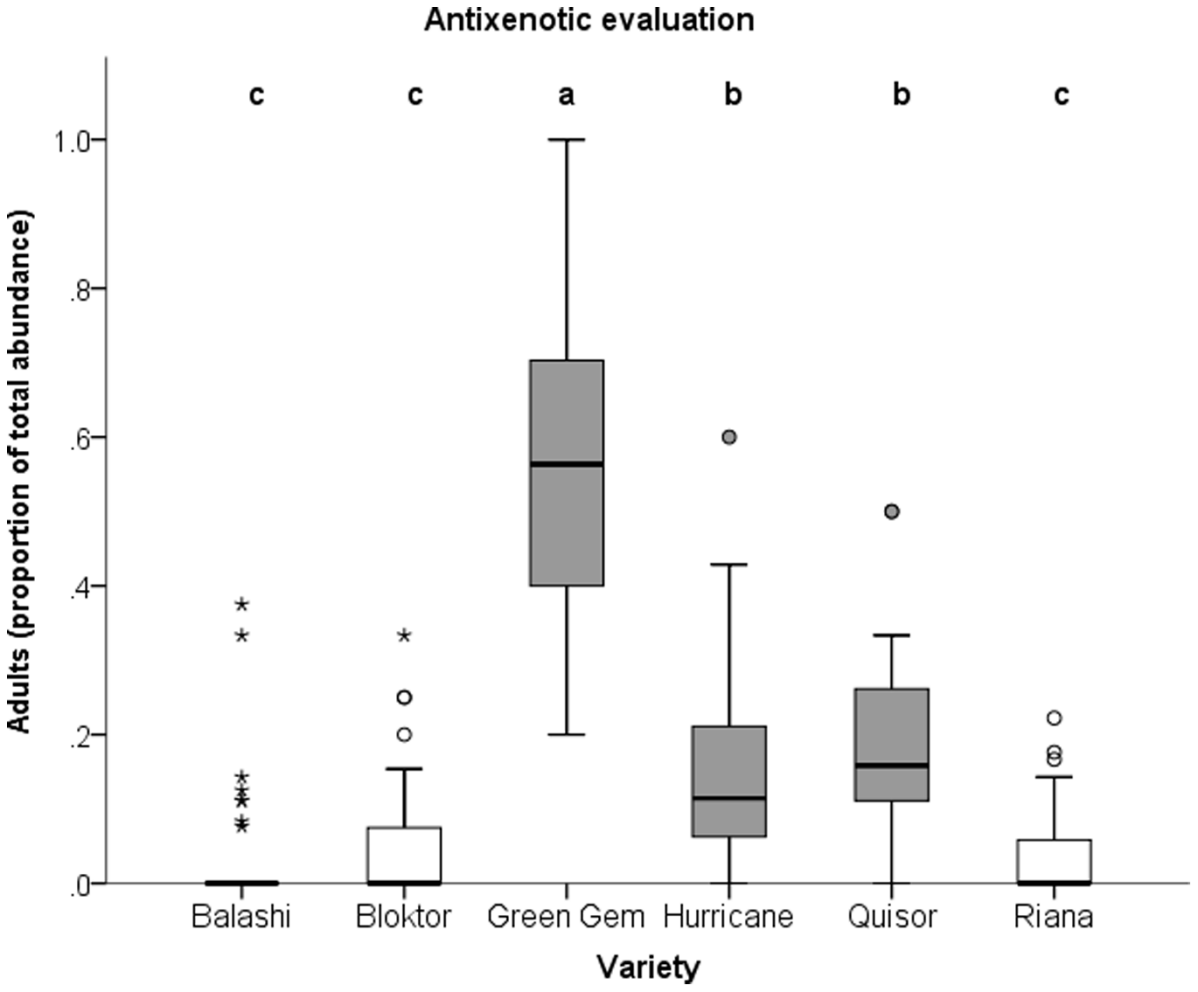
Antixenotic evaluation in the early stage of white cabbage head formation. Significance: Means with different letters are significantly different from each other (p≤0.01, Mann-Whitney U test).

Statistics revealed that here was no varietal block effect (p = 0.13). The AR assessment yielded significant differences between the studied cabbage varieties Significantly more thrips adults were found on susceptible varieties (Green Gem, Quisor and Hurricane) than on those varieties (Bloktor, Riana and Balashi) that were categorized resistant in previous studies [12] (Figure 1). The Spearman’s rank correlation between number of thrips and weight of cabbage revealed no significant positive correlation (n = 288, rho = −0.116, p = 0.09).

### Leaves Reflection

**Figure 2:**
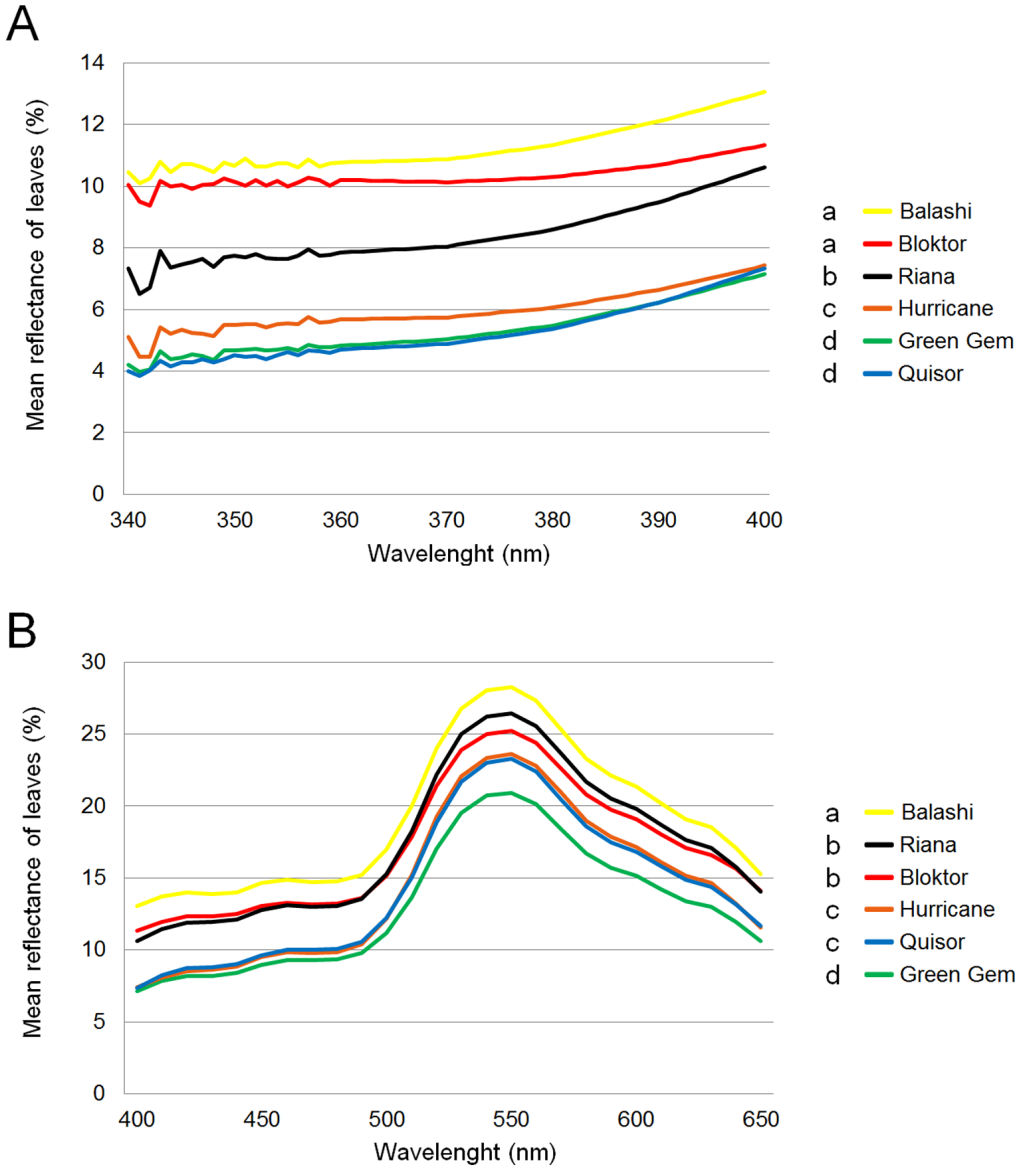
Reflection of head forming leaves in UV-A (A) and visible range (B) in the early period of the white cabbage head formation. Explanation: For data analyses the reflection was measured in 12 leaves four times for each variety from 340 nm to 650 nm. These four values were averaged (n = 12×average of 4 measurements/each variety). An average reflection value for each nm for UV-A (340–400 nm) were computed for each leaf and a sum of 12 average reflection values were obtained for each variety. These served as quantitative variable for statistical analyses (A). The same approach between 400 to 650 nm was used to get values for visible range (B). Balashi, Bloktor and Riana are considered resistant, Green Gem, Hurricane and Quisor are considered susceptible to onion thrips. Significance: Means with different letters are significantly different from each other (UV-A with Games-Howell test and visible range of light by Tukey HSD test).

**Figure 3:**
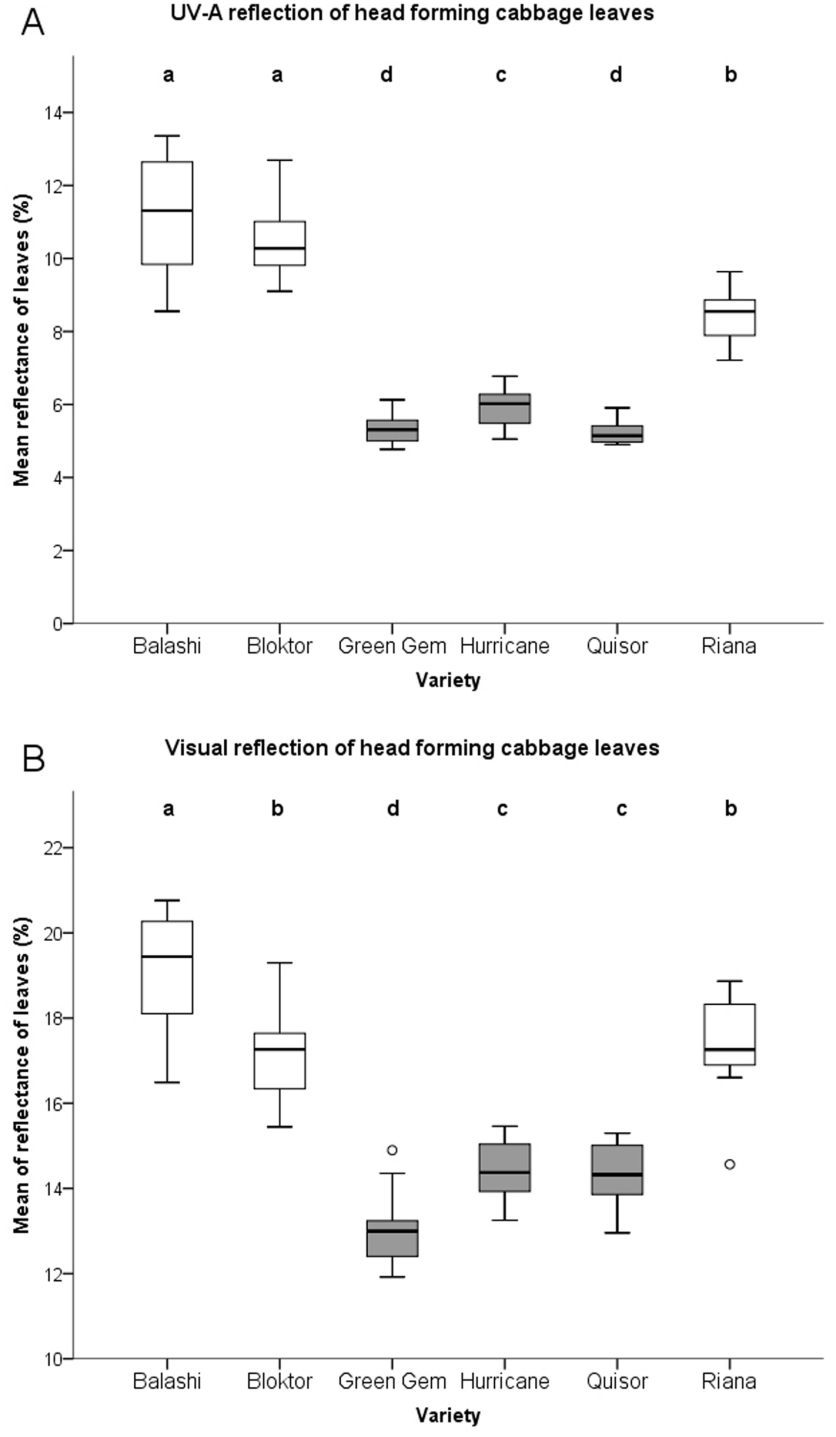
Reflection of head forming cabbage leaves in UV-A (A) and visible range (B). Explanation: For data analyses the reflection was measured in 12 leaves four times for each variety from 340 nm to 650 nm. These four values were averaged (n = 12×average of 4 measurements/each variety). An average reflection value for each nm for UV-A (340–400 nm) were computed for each leaf and a sum of 12 averages of reflection values were obtained for each variety. These served as quantitative variable for statistical analyses (A). Significance: Means with different letters are significantly different from each other (UV-A with Games-Howell test and visible range of light by Tukey HSD test).

**Figure 4:**
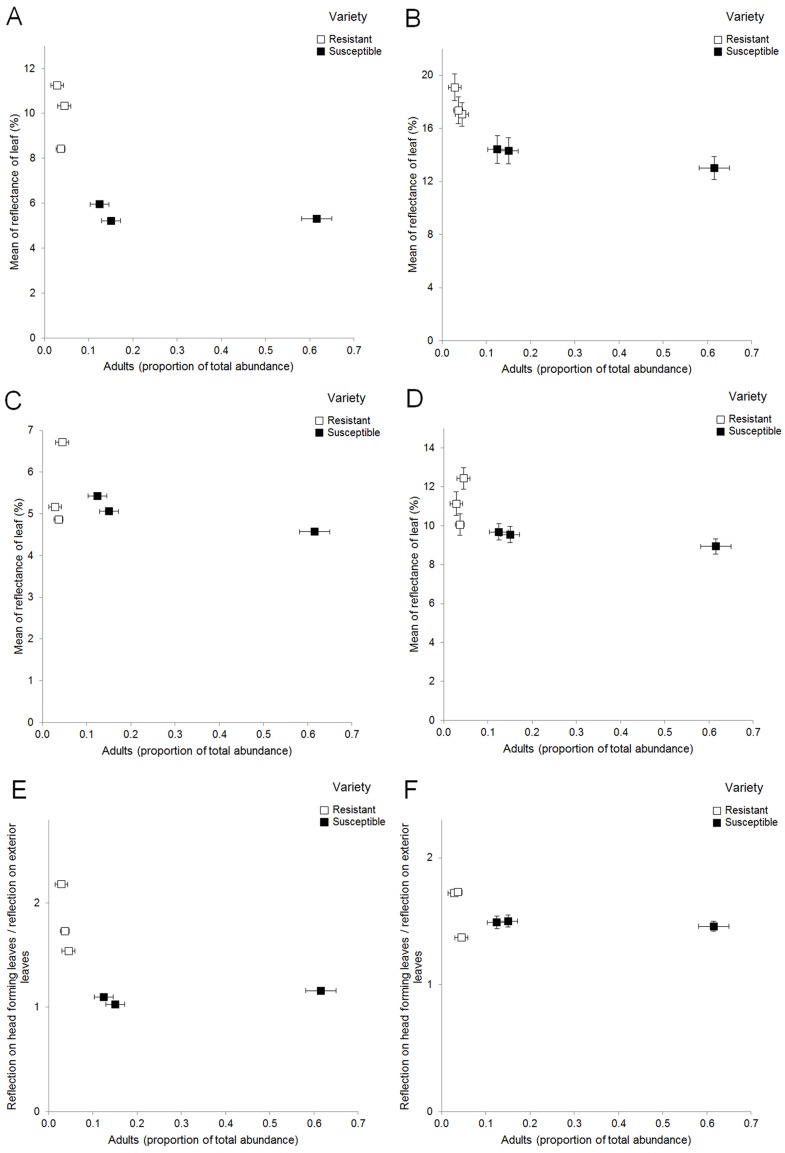
Plots between mean reflection (UV-A and visible) and antixenotic resistance values. A - UV-A reflection on head forming leaves and PTA, B - Visual reflection on head forming leaves and PTA, C - UV-A reflection on exterior leaves and PTA, D - Visual reflection on exterior leaves and PTA, E - Contrast UV-A reflection and PTA, F - Contrast Visual reflection and PTA.

Statistics revealed no varietal block effect in UV-A reflection of head forming leaves (p = 0.83) and visible reflection of head forming leaves (p = 0.61). Significant differences in UV-A reflection of head forming leaves were observed between resistant varieties and also between resistant and susceptible ones (Figure 2A, Figure 3A). The mean visible reflection was also different between resistant and susceptible varieties (Figure 2B, Figure 3B). Negative correlation was detected between UV-A reflection of head forming leaves and PTA, as well as between the UV-A brightness contrast of exterior and head forming leaves and PTA in the early stage (34–114 g) of white cabbage head formation. The mean visible reflection of both head forming and exterior leaves was also in negative correlation with mean PTA in the early stage of the cabbage head development. No correlation between UV-A reflection of exterior leaves only and PTA or between contrast in visible range and PTA were detected (Table 2, Figure 4 A–F).

**Table 2:** Spearman’s rank correlation between mean reflection (UV-A and visible) and proportion of total abundance (PTA) values (n = 6).

Spearman’s correlation between UV-A and PTA				
Head forming leaves	Exterior leaves	Contrast H/E		
rho	**−0.886**	−0.371	**−0.829**	
Sign	**0.019**	0.468	**0.042**	
Spearman’s correlation between visible range and PTA				
rho	**−1.000**	**−0.829**		−0.543
Sign	**0.01**	**0.042**		0.266

Significances are in bold.

### Onion Thrips Damage Assessment at Harvest

Statistics revealed no varietal block effect in thrips damage (p = 0.87) and number of damaged leaves (p = 0.82). Hurricane was damaged the most by thrips considering both the total number of damaged leaves and the extent of damaged leaf surface. Green Gem and Quisor also suffered significantly greater damage than any of the resistant varieties. Considerably less damage was observed on resistant varieties (Bloktor, Balashi and Riana) (Table 3). Highly positive correlation was observed between number of adult thrips and damage at harvest maturity (n = 288, Spearman’s rho = 0.541, p<0.001).

**Table 3:** Damage assessment at harvest considering the mean number of damaged leaves and total damaged leaf surface.

Number of damaged leaves					Total damaged leaf surface (1,0 = 1 leaf surface)				
Variety	Date of assessment	Mean^c^	a^a^	95%Conf.^b^	Variety	Date of assessment	Mean^c^	a^a^	95%Conf.^b^
Hurricane	17/09	14.6	a	1.2	Hurricane	17/09	4.0	a	0.3
Green Gem	12/08	8.8	b	0.8	Green Gem	12/08	2.1	b	0.3
Quisor	19/08	6.8	c	0.5	Quisor	19/08	1.6	c	0.2
**Bloktor**	10/10	4.4	d	0.5	**Bloktor**	10/10	0.8	d	0.1
**Balashi**	11/08	1.0	e	0.2	**Balashi**	11/08	0.1	e	0.0
**Riana**	21/08	0.5	f	0.2	**Riana**	21/08	0.0	f	0.0

a Significance: Means with different letters are significantly different from each other (p≤0.01, Mann-Whitney U test).

b 95%-confidence interval of means.

c Means are presented in decreasing order.

Resistant varieties are in bold.

## Discussion and Conclusion

Results in concordance with other studies support the idea that the antixenotic resistance of white cabbage against a polyphagous pest such as onion thrips does exist. The onion thrips density was always higher on susceptible (Green Gem, Hurricane, Quisor) than on resistant varieties (Balashi, Bloktor, Riana). Similar studies evaluated the number of thrips on both exterior and head forming leaves [25], but we only made assessment on head forming leaves because these individuals are responsible for damage that appears as a consequence of the plant’s reaction to thrips feeding. Colonization from exterior leaves however may be possible before thrips become trapped by head leaves. Altogether the antixenotic evaluation based on colonizing thrips adults at the beginning of cabbage head formation can be considered an appropriate method of comparing different white cabbage varieties.

Results also suggest that thrips numbers and damage were differentially associated with different reflectance values. Reflectance of white cabbage leaves may be defined as one important physical factor that plays a role in AR. Kirk [26] found similar results using color traps and reported that UV reflecting white color traps were ten times less attractive to onion thrips than white traps without UV reflectance. The phenomenon was also observed on one other thrips species too, where the repelling effect of UV reflecting metalized mulch was used against *Frankliniella occidentalis* (Pergande, 1895) [27]. Cabbage plants surrounded by UV reflecting metalized mulch in comparison with standard black soil coverage were less attractive for thrips [27]. In our studies negative correlation was observed between PTA and UV-A reflection and visual reflection of head forming leaves. Also negative correlation was detected between PTA and UV-A contrast and visible reflection of both head forming and exterior leaves in the early stage of white cabbage head formation (Table 2). These results altogether suggest that AR against onion thrips may be at least partly determined by differences in reflection of cabbage leaves at an early growth stage. The lower reflectance of head forming leaves alone or in contrast with brighter exterior leaves may attract onion thrips to invade the head leaves of susceptible varieties to a greater extent.

Similar studies reported strong positive correlation between the colonization of adult thrips and the damage ratings [12]. We can confirm this with the present work, observing that susceptible varieties suffered higher damage at harvest than resistant ones and again highly positive correlations were observed between number of adult thrips and damage.

In summary we can conclude that white cabbage leaves’ UV and visible reflection might play a role in attracting thrips to colonize cabbage head leaves and as a consequence these thrips individuals cause direct damage.
