# Correction: Correlations between Colonization of Onion Thrips and Leaf Reflectance Measures across Six Cabbage Varieties

**DOI:** 10.1371/annotation/225fb043-04ec-4bc1-91d7-ed4c49ab3dc7

**Published:** 2013-09-18

**Authors:** János Bálint, Balázs Vince Nagy, József Fail

There was an error in the Funding statement. The correct version of the statement is available below.

This study was financially supported by the Balassi Institute - Hungarian Scholarship Board Office and Institute for Ethnic and National Minority Studies of the Hungarian Academy of Sciences. Bakker Brothers, Bejo Zaden B.V., Daehnfeldt A/S and Syngenta Seeds Inc. are acknowledged for providing seeds for this study. Author BVN was supported by FAPESP (09/54292-7). The publication of this study was supported by the grant TAMOP 4.2.2/B-10/1-2010-0023. The funders had no role in study design, data collection and analysis, decision to publish, or preparation of the manuscript. 

